# Leanness

**Published:** 1892-01

**Authors:** 


### LEANNESS.

Some persons are born with a normal tendency to become fat, others with a tendency to leanness. It is the same among the lower animals. The hog is a sort of machine for transforming the odds and ends of food into fat ; but the farmer knows beforehand that a little pig with long legs and snout will work off the fat as fast as it can be made. So a long-legged person seldom inclines to obesity. Temperament has much to do with the bodily condition in this respect. In lymphatic people the life processes are slow, and the fat is largely deposited rather than burned. This temperament furnishes some of the best types of surface beauty. The person of nervous temperament, on the other hand, by excessive activity of body and mind, and by predisposition to haste, worry, fret and impatience, naturally remains lean ; but while the features of such a person will probably lack softness and roundness of outline, they may exhibit in a marked degree the higher beauties of mind and soul. People who incline to obesity may hold the tendency in check by appropriate food and stirring exercise in the open air, thus both lessening the amount of fat forming food taken into the system, and causing a more rapid consumption of such fat as is produced ; and those who incline to undue leanness, by pursuing the opposite course, may largely increase the amount of fat deposited. If the leanness is the result of digestive weakness, or of a faulty assimilation, little, of course, can be done until a condition of general health has been secured. But assuming that the abnormal leanness is connected with high health, the carbonaceous, or fat-forming, food should greatly preponderate over the nitrogenous—such as beef, lamb and codfish. Calling the fat forming elements of beef 20, lamb 35, and codfish 5, those of pork will be 50 ; beans, 57 ; peas, 60 ; oats, 66 ; wheat, 69 ; corn and rye, each 72 ; rice, 80 ; and butter, 100. Of course it would not do to take a single carbonaceous article and live on it, for the entire body is to be kept in high health by the proper nourishment of all the tissues. However, the system can be well supported in full vigor by a vegetable diet, with the addition of milk, eggs and butter. Cultivate calmness and quietness in feeling and manner. Avoid impatience and fret. Do not overwork with mind or body. Tea drinking tends to leanness; if possible, milk should be substituted.

